# Dosimetric divergence in ICBT vs. IC/ISBT configurations: Comparative analysis of three optimization algorithms for cervical cancer brachytherapy

**DOI:** 10.1371/journal.pone.0335405

**Published:** 2025-11-13

**Authors:** Jihong Chen, Jiabiao Hong, Kaiqiang Chen, Xiuchun Zhang, Guohua Wang, Penggang Bai

**Affiliations:** 1 Department of Radiation Oncology, Clinical Oncology School of Fujian Medical University, Fujian Cancer Hospital, Fuzhou, Fujian, China; 2 School of Nuclear Science and Technology, University of South China, Hengyang, China; All India Institute of Medical Sciences, INDIA

## Abstract

**Objective:**

To compare dosimetric differences among graphical-based manual planning (MA), simulated annealing inverse optimization (IPSA), and hybrid inverse optimization (HIPO) for cervical cancer in both intra-cavitary brachytherapy (ICBT) and interstitial brachytherapy combined with ICBT (IC/ISBT) settings, providing evidence for clinical optimization method selection.

**Methods:**

This study consisted of 60 cervical cancer patients undergoing CT-guided three-dimensional brachytherapy, including 30 ICBT patients and 30 IC/ISBT patients. Plans were generated using MA, IPSA, and HIPO. The dosimetric parameters for the high-risk clinical target volume (HRCTV) including D_100%_, V_150%_, V_200%_, conformity index (CI), homogeneity index (HI) were compared. Meanwhile, the dosimetric parameters D_1cc_, D_2cc_ for the bladder, rectum, sigmoid, and total treatment time were evaluated.

**Results:**

Compared with MA, both IPSA and HIPO delivered lower doses to organs at risk (OARs). The total treatment time was significantly shorter for HIPO compared to IPSA and MA (P < 0.05). In ICBT patients, the D_1cc_ and D_2cc_ of OARs were lower for IPSA compared to HIPO (P > 0.05), while the CI was significantly better for HIPO (P < 0.05). Nevertheless, in IC/ISBT patients, D_2cc_ of rectum for HIPO was significantly lower compared to IPSA (P < 0.05), with better CI.

**Conclusion:**

Inverse optimization effectively reduces doses to OARs while maintaining target coverage. HIPO appears to be the preferred choice for IC/ISBT, due to shortened treatment time, superior CI and rectal protection compared with IPSA.

## 1 Introduction

Cervical cancer is a common gynecological malignancy significantly threatening women’s health globally [1]. Brachytherapy (BT), which delivers radiation directly into or near tumors, is crucial in cervical cancer treatment [2,3]. Intra-cavitary brachytherapy (ICBT), using applicators inserted through natural body cavities to deliver radiation, is a relatively simple and minimally invasive technique and constitutes the standard approach for BT [4]. However, when tumor volume is large, regression is inadequate, or vaginal stenosis occurs, ICBT alone may yield suboptimal target coverage. In such cases, interstitial brachytherapy (ISBT) combined with ICBT (IC/ISBT) is employed to enhance target coverage [5,6].

Traditional forward planning depends on physicists manually adjusting dwell positions and times for target coverage and organs at risk (OARs) protection. Graphical optimization (Gro), a typical forward optimization method, manually adjusts isodose lines to cover targets and spare adjacent normal tissues. Over the past two decades, inverse planning has gained popularity due to shorter planning times, better repeatability, higher efficiency, potentially superior target coverage, and reduced OARs dose [7–17]. Inverse Planning Simulated Annealing (IPSA) [18], which optimizes dwell times based on anatomical structures and defined constraints, is widely used due to its efficacy and popularity. Christopher et al. found that high-dose-rate (HDR) BT planned using IPSA was well tolerated and provided excellent local control [19]. More recently, Hybrid Inverse Planning Optimization (HIPO), which combines simulated annealing and limited-memory deterministic algorithms (L-BFGS), offers three-dimensional dose distribution and allows for manual source channel locking, representing an advanced volumetric optimization tool [20]. Petra et al. demonstrated that HIPO can generate clinically acceptable treatment plans and eliminate high-dose regions in normal tissue [21].

Studies have found that HIPO provides prostate BT plans clinically comparable to IPSA, with enhanced conformity, potentially more homogeneous dwell times, and reduced hotspots [22]. Similarly, IPSA and HIPO have been shown to achieve similar dosimetry for interstitial tongue HDR BT [23]. However, comparative studies of HIPO and IPSA for cervical cancer BT, especially involving different applicators, remain limited [24,25] . These related articles typically compare IPSA and HIPO primarily in single scenarios such as IC/ISBT or ICBT, without directly comparing them with the most commonly used graphical optimization algorithms in clinical practice. In contrast, this study compares dosimetric outcomes and dwell time distribution characteristics among Manual (MA), IPSA, and HIPO-generated BT plans for both IC/ISBT and ICBT scenarios, within the same institution under consistent data conditions and offering more practical clinical references.

## 2 Materials and methods

### 2.1 Clinical data

This study was approved by the Ethics Committee of Fujian Provincial Cancer Hospital (K2022-184–01), and patient informed consent was waived. All studies adhered to the Declaration of Helsinki. Sixty cervical cancer patients receiving three-dimensional BT between December 2024 and April 2025 at Fujian Provincial Cancer Hospital were retrospectively enrolled, with 30 patients each in the ICBT and IC/ISBT groups. Collected images were fully anonymized, preventing author access to personally identifiable information. Patient ages ranged from 38 to 82 years, with a mean age of 65 years and a median of 67 years. All patients had histologically confirmed diagnoses, complete medical records, and good tolerance for radiotherapy. The initial BT treatment plan for each patient was included in the analysis.

### 2.2 Applicator implantation and CT scanning

Patients were placed in the lithotomy positions. Oral contrast medium (iopamidol) was administered to delineate the small intestine, and 100 ml of saline solution was infused into the bladder before each CT scan. Nucletron standard tandem and ovoid applicators were used in the ICBT group, and tandem applicators combined with four interstitial needles were employed in the IC/ISBT group. CT images were acquired using a Philips Brilliance CT Big Bore scanner (Philips Medical Systems Inc., Cleveland, OH, USA) with a 512 × 512-pixel matrix and 2.5 mm slice thickness.

### 2.3 Planning design

CT images were transferred to the Oncentra Brachy planning system (version 4.6.3, Elekta, Stockholm, Sweden). Physicians delineated HRCTV and OARs following GEC-ESTRO guidelines, including the lower uterus, cervix, parametrium, and upper vagina. OARs included bladder, rectum, and sigmoid. After reconstructing applicator channels on original CT images, three plans were created for each patient using the three optimization methods. Continuous plan adjustments ensured the dose corresponding to 90% (D_90_) of HRCTV volume reached 6 Gy (within 0.01 Gy) while minimizing radiation dose to OARs. The source activity was set to 8 Ci.

The MA group used manual dwell position selection and graphical optimization to adjust isodose lines.

IPSA plans automatically defined dwell positions, setting constraints for minimal target surface dose and maximal OAR surface dose (Table 1). For IPSA, a dwell time deviation constraint (DTDC) [26] of 0.5 was used to prevent isolated dwell positions with excessively long times.

**Table 1:** Dose volume optimization parameters for IPSA and HIPO plan.

	OAR (surface)	Weight	Min (Gy)	Max (Gy)	Weight
IPSA	HRCTV	100	6		
	Bladder			3.5	90
	Rectum			3	80
	Sigmoid			3	80
HIPO	HRCTV	100	6	14	1
	Bladder			3.5	90
	Rectum			3	80
	Sigmoid			3	80
	Normal tissue			6	50

HIPO plans added maximum dose constraints to HRCTV (Table 1). For HIPO, a dwell time gradient restriction (DTGR) [27] of 0.5 was applied to limit fluctuations between neighboring dwell times, consistent with prior studies. In order to reduce the planning time, both IPSA and HIPO plans were manually fine-tuned post-optimization when D_90_ of HRCTV was very close to 6Gy (within 0.05 Gy).

### 2.4 Dosimetric evaluation

Planning time and treatment times were recorded, and several dosimetric parameters were analyzed using dose-volume histograms (DVHs). HRCTV dose corresponding to 100% of volume (D_100_), the volume percentage of 100%, 150% and 200% of the prescription dose (V_100%_, V_150%_, V_200%_), conformity index (CI=(V_prescription in HRCTV_/V_HRCTV_)*(V_prescription in HRCTV_/V_prescription_)) and homogeneity index ((HI=(V_100%_-V_150%_)/V_100%_)) were all evaluated. Meanwhile, Dose corresponding to 1cc volume (D_1cc_) and 2cc volume (D_2cc_) of bladder, rectum and sigmoid were calculated.

### 2.5 Statistical analysis

Paired t-tests (for normally distributed data) or Wilcoxon signed-rank tests (for non-normally distributed data) were carried out for dosimetric parameters previously described. Statistical package for the Social Sciences (SPSS 21.0; SPSS Inc., Chicago, IL, USA) was used to perform these tests and p < 0.05 was considered statistically significant. (Dosimetric comparison with the Benjamini-Hochberg false discovery rate correction of MA, IPSA and HIPO plans in the ICBT cohort and the IC/ISBT cohort were listed separately in S1 and S2 Tables.)

## 3 Results

The HRCTV volume among the 60 patients ranged from 17.7 to 86.7 cm³ (median 42.6 cm³). Median volumes were 40.1 cm³ in the ICBT cohort and 43.4 cm³ in the IC/ISBT cohort.

### 3.1 ICBT cohort

**Fig 1:**
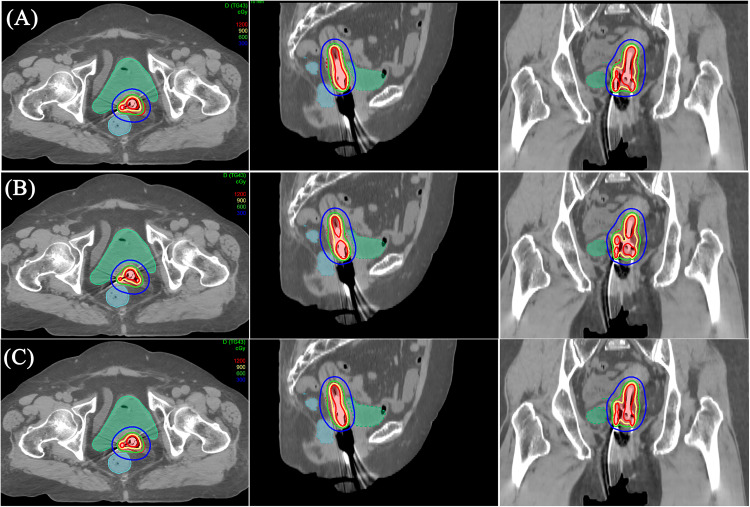
Dose distribution for each plan for a patient in ICBT cohort. (A): MA. (B): IPSA. (C): HIPO.

Fig 1 shows the dose distribution of MA, IPSA and HIPO for the same patient in the ICBT cohort. The high-dose region (9 Gy and 12 Gy) of IPSA was smaller than that of MA and HIPO.

**Fig 2:**
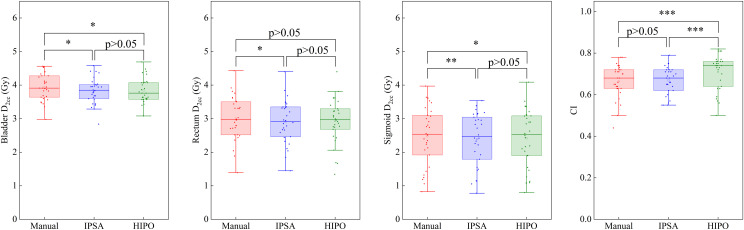
Box plots of the main dosimetric parameters (D_2cc_ for bladder, rectum and sigmoid, CI) for the MA, IPSA and HIPO plans in the ICBT cohort. *: p-value between 0.01 and 0.05. **: p-value between 0.001 and 0.01. ***: p-value≤0.001.

IPSA plans yielded significantly lower V_150%_ and V_200_% values and a higher HI than MA plans (p < 0.05), as shown in Table 2 and Fig 2. HIPO produced the best CI, superior to both MA and IPSA plans (p < 0.05). For OARs, the doses in IPSA and HIPO plans were consistently below those of MA plans; differences were significant except for rectum D_1cc_ and D_2cc_ in HIPO (p > 0.05). Meanwhile, the OAR doses for IPSA and HIPO were comparable and showed no statistically significant difference.

**Table 2:** Dosimetric comparison of Manual, IPSA and HIPO plans in the ICBT cohort (mean ± SD).

	Parameter	MA	IPSA	HIPO	P1	P2	P3
HRCTV	D_100_ (Gy)	3.55 ± 0.40	3.61 ± 0.45	3.52 ± 0.42	0.418	0.682	0.278
	V_150%_ (%)	53.07 ± 3.70	51.66 ± 2.45	52.03 ± 3.68	0.030^*^	0.091	0.523
	V_200%_ (%)	31.19 ± 3.33	29.82 ± 2.28	30.49 ± 3.38	0.010^*^	0.145	0.156
	HI	0.41 ± 0.04	0.43 ± 0.03	0.42 ± 0.04	0.030^*^	0.095	0.505
	CI	0.66 ± 0.08	0.67 ± 0.06	0.71 ± 0.08	0.558	0.000^*^	0.000^*^
Bladder	D_1cc_ (Gy)	4.25 ± 0.44	4.10 ± 0.39	4.15 ± 0.39	0.006^*^	0.021^*^	0.214
	D_2cc_ (Gy)	3.94 ± 0.40	3.83 ± 0.38	3.84 ± 0.37	0.017^*^	0.010^*^	0.646
Rectum	D_1cc_ (Gy)	3.37 ± 0.72	3.25 ± 0.69	3.29 ± 0.72	0.010^*^	0.102	0.299
	D_2cc_ (Gy)	3.00 ± 0.66	2.91 ± 0.64	2.93 ± 0.66	0.019^*^	0.077	0.546
Sigmoid	D_1cc_ (Gy)	2.78 ± 0.98	2.67 ± 0.87	2.71 ± 0.97	0.007^*^	0.045	0.298
	D_2cc_ (Gy)	2.48 ± 0.86	2.39 ± 0.79	2.41 ± 0.85	0.009^*^	0.026^*^	0.450

P1 represents p-value of IPSA vs. MA; P2 represents p-value of HIPO vs. MA; P3 represents p-value of IPSA vs. HIPO. All p-values derived from paired t-tests.

*: p-value < 0.05.

### 3.2 IC/ISBT cohort

**Fig 3:**
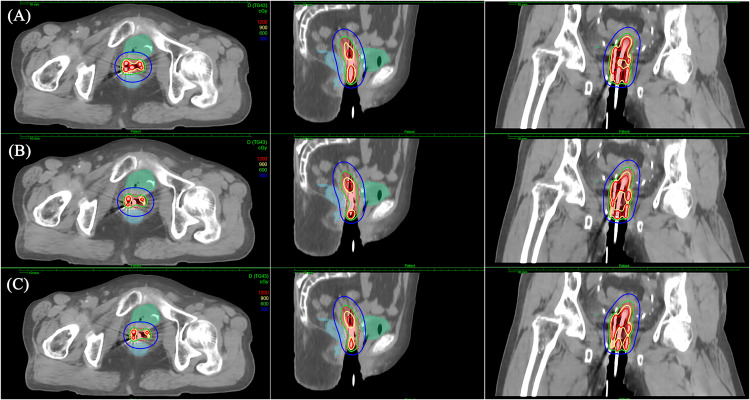
Dose distribution for each plan for a patient in IC/ISBT cohort. (A): MA. (B): IPSA. (C): HIPO.

Fig 3 shows the dose distribution of MA, IPSA and HIPO for the same patient in the IC/ISBT cohort. The high-dose region (9 Gy and 12 Gy) of MA was larger than that of IPSA and HIPO, especially in the tandem region.

**Fig 4:**
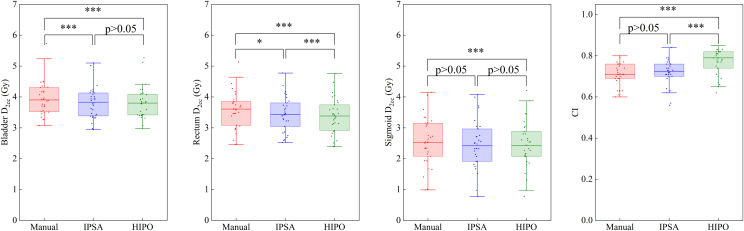
Box plots of the main dosimetric parameters (CI, D_2cc_ for bladder, rectum and sigmoid) for the MA, IPSA and HIPO plans in the IC/ISBT cohort. *: p-value between 0.01 and 0.05. **: p-value between 0.001 and 0.01. ***: p-value≤0.001.

Both IPSA and HIPO plans showed significantly lower V_150%_ and V_200_% and higher HI and CI than MA plans (all p < 0.001), as shown in Table 3 and Fig 4. HIPO achieved the highest CI, outperforming IPSA (p < 0.001). OAR doses in IPSA and HIPO were below MA values. Rectum D_1cc_ in IPSA did not differ from MA, whereas HIPO yielded significantly lower rectum D_1cc_ and D_2cc_ than IPSA.

**Table 3:** Dosimetric comparison of Manual, IPSA and HIPO plans in the IC/ISBT cohort (mean ± SD).

	Parameter	MA	IPSA	HIPO	P1	P2	P3
HRCTV	D_100_ (Gy)	3.46 ± 0.31	3.53 ± 0.34	3.56 ± 0.28	0.224	0.094	0.595
	V_150%_ (%)	52.22 ± 4.66	47.84 ± 5.47	47.4 ± 6.14	0.000^*^	0.000^*^	0.299
	V_200%_ (%)	27.93 ± 5.47	25.66 ± 4.93	25.06 ± 5.49	0.000^*^	0.000^*^	0.083
	HI	0.42 ± 0.05	0.47 ± 0.06	0.47 ± 0.07	0.000^*^	0.000^*^	0.300
	CI	0.71 ± 0.05	0.72 ± 0.06	0.77 ± 0.06	0.443	0.000^*^	0.000^*^
Bladder	D_1cc_ (Gy)	4.28 ± 0.64	4.05 ± 0.55	4.09 ± 0.55	0.000^*^	0.000^*^	0.074
	D_2cc_ (Gy)	3.96 ± 0.57	3.80 ± 0.52	3.81 ± 0.52	0.000^*^	0.000^*^	0.613
Rectum	D_1cc_ (Gy)	3.99 ± 0.72	3.80 ± 0.65	3.75 ± 0.69	0.647	0.000^*^	0.000^*^
	D_2cc_ (Gy)	3.58 ± 0.62	3.45 ± 0.56	3.37 ± 0.59	0.022^*^	0.000^*^	0.000^*^
Sigmoid	D_1cc_ (Gy)	2.86 ± 0.86	2.76 ± 0.87	2.73 ± 0.87	0.034^*^	0.001^*^	0.348
	D_2cc_ (Gy)	2.55 ± 0.79	2.47 ± 0.79	2.44 ± 0.78	0.047^*^	0.001^*^	0.261

P1 represents p-value of IPSA vs. MA; P2 represents p-value of HIPO vs. MA; P3 represents p-value of IPSA vs. HIPO. All p-values derived from paired t-tests.

*: p-value≤0.05.

### 3.3 Plan characteristics

HIPO markedly shortened total dwell time compared with MA and IPSA plans in both cohorts (p < 0.05), shown in Table 4. IPSA devoted the smallest proportion of dwell time to the tandem, followed by HIPO. The mean optimization time for IPSA was the shortest, followed by HIPO and MA.

**Table 4:** Dwell-time metrics and optimization time for MA, IPSA and HIPO plans (mean ± SD).

	Parameter	MA	IPSA	HIPO	P1	P2	P3
ICBT	Total loading time (s)	325.34 ± 182.27	324.92 ± 183.99	307.93 ± 177.84	0.704	0.000	0.000
	T_tan/tot_	0.56 ± 0.15	0.50 ± 0.15	0.53 ± 0.15	0.005	0.069	0.136
	Optimization time (s)	122.57 ± 19.02	16.47 ± 4.02	19.26 ± 3.82	0.000	0.000	0.006
IC/ISBT	Total loading time (s)	271.63 ± 66.24	270.15 ± 56.53	256.27 ± 56.29	0.517	0.000	0.000
	T_tan/tot_	0.48 ± 0.16	0.44 ± 0.16	0.46 ± 0.14	0.000	0.000	0.275
	Optimization time (s)	128.90 ± 17.69	16.06 ± 2.91	19.36 ± 5.01	0.000	0.000	0.003

P1 represents p-value of IPSA vs. MA; P2 represents p-value of HIPO vs. MA; P3 represents p-value of IPSA vs. HIPO. All p-values derived from Wilcoxon signed-rank test. T_tan/tot_ represents the ratio of tandem loading time in the total loading time.

## 4 Discussion

Three-dimensional BT planning can use forward methods (e.g., Gro) or inverse algorithms (e.g., IPSA, HIPO). Forward planning is labour-intensive, highly operator-dependent, and sometimes fails to achieve an acceptable dose distribution in complex cases. Inverse planning leverages computational optimization to satisfy target and OAR objectives more efficiently. In this study, we simultaneously explored the dosimetric differences resulting from three planning methods under both IC/ISBT and ICBT scenarios. Our results indicate that under ICBT, the differences in organ doses among the three plans are relatively small. In contrast, under IC/ISBT, HIPO demonstrates lower rectal doses, which may reduce the probability of rectal toxicities. Additionally, HIPO achieves the shortest treatment time across all scenarios. Although its planning time is slightly longer than that of IPSA, the overall time remains the lowest, which could improve the daily workflow efficiency and reduce patient discomfort.

In the present study, all three approaches met prescription requirements. For ICBT, with fewer applicator channels, the high-dose volumes and homogeneity achieved by IPSA and HIPO were largely comparable with MA, though MA relied more heavily on tandem dwell times, producing larger high-dose regions that remain clinically tolerable and desirable. The organ doses for IPSA and HIPO are generally comparable and are significantly lower than those of MA, consistent with previous findings [28,29]. When applicator geometry became more complex in IC/ISBT, HIPO demonstrated certain advantages. It significantly decreased rectal dose and improved CI compared with IPSA while maintaining similar doses to other OARs. In this study, the coverage of HRCTV for all plans were set to 90%. Moreover, there is no clear clinical evidence demonstrating that plans with better HI are associated with superior clinical outcomes [30]. Therefore, it can be concluded that the tumor control probability (TCP) is comparable among the three plans. A larger CI implies a smaller volume of prescription dose delivered to normal tissues. A radiobiological study about cervical cancer BT reported that an increase of 0.11 Gy in rectum D_2cc_ (from 4.26 Gy to 4.37 Gy) raised the normal tissue complication probability (NTCP) of rectum from 1.62% to 2.15%. Similarly, an increase of 0.18 Gy in bladder D_2cc_ (from 4.59 Gy to 4.77 Gy) increased the NTCP of bladder from 0.23% to 0.24% [31]. The difference in D_2cc_ for OARs between inverse planning and MA were approximately on the order of 0.10–0.2 Gy in our study, which could affect the NCTP. Additionally, another multicenter prospective cohor study indicated that a rectum D_2cc_ < 65 Gy, summed BT doses with external beam radiotherapy doses and converted to 2Gy per fraction equivalent dose (EQD2), was associated with a two times lower risk of proctitis than D_2cc_ ≥ 65Gy [32]. In the context of IC/ISBT, the difference in rectum EQD2 (α/β = 3 Gy, in 5 fractions) between HIPO and MA reached 2.1 Gy (23.6Gy vs. 21.5Gy), which is approximately 3% of the threshold dose and quite considerable.

The most significant difference between HIPO and IPSA optimization lies in the distribution of source residence time. Spatial dwell-position distribution was more continuous and even with HIPO, whereas IPSA exhibited larger dwell-time fluctuations [33]. When the residence time at a certain spatial position within the channel varies greatly, it may lead to local dose hotspots or insufficient doses in the target area, and even cause dose limits to be exceeded for OARs. HIPO also shortened total irradiation time by roughly 5% for both cohorts, a benefit that may enhance applicator stability between fractions and reduce patient discomfort. Therefore, in the planning design of cervical cancer IC/ISBT, the HIPO optimization algorithm can serve as an effective alternative to IPSA.^31^ In a study by Pooriwat et al., it was found that time delays during both treatment planning and delivery can impact OAR doses, as additional time may lead to increased urine accumulation in the bladder [34]. Therefore, it is crucial to minimize the overall procedural duration. In this study, when considering both planning and delivery times, HIPO required significantly less time compared to IPSA and MA. Moreover, it is worth noting that as source activity decreases, the difference in overall time becomes more pronounced. This advantage may contribute to enhancing the BT workflow efficiency.

Of course, this study also has some limitations. First, this study is solely a retrospective dosimetric comparison and lacks relevant clinical data. Second, constrained by the number of cases, the study only considered the most common four-needle configuration at our center. Therefore, the applicability of the findings from this study to cases with different numbers of interstitial needles remains to be validated. Third, to facilitate comparison, the DTGR constraint for HIPO and the DTDC for IPSA were both set to a fixed value of 0.5. The selection of this parameter could influence the final result of the inverse optimization and warrants further investigation in subsequent studies [35].

## 5 Conclusion

Both IPSA and HIPO inverse-planning algorithms achieve prescription coverage while lowering OAR doses compared with MA. In more complex IC/ISBT setting, HIPO should be the first-line strategy, due to further lower rectal dose and shorter treatment time.

## Supporting information

S1 Table Dosimetric comparison with the Benjamini-Hochberg false discovery rate correction of MA, IPSA and HIPO plans in the ICBT cohort (mean ± SD). P1 represents p-value of IPSA vs. MA; P2 represents p-value of HIPO vs. MA; P3 represents p-value of IPSA vs. HIPO. *: p-value≤0.05. All p-values were derived from paired t-tests and adjusted for multiple comparisons using the Benjamini-Hochberg (FDR) correction.
(DOCX)

S2 Table Dosimetric comparison with the Benjamini-Hochberg false discovery rate correction of MA, IPSA and HIPO plans in the IC/ISBT cohort (mean ± SD). P1 represents p-value of IPSA vs. MA; P2 represents p-value of HIPO vs. MA; P3 represents p-value of IPSA vs. HIPO. *: p-value≤0.05. All p-values were derived from paired t-tests and adjusted for multiple comparisons using the Benjamini-Hochberg false discovery rate (FDR) correction.
(DOCX)
