# Supplementary material for: Dosimetric divergence in ICBT vs. IC/ISBT configurations: Comparative analysis of three optimization algorithms for cervical cancer brachytherapy
Source: PLoS One. 2025 Nov 13;20(11):e0335405. doi: 10.1371/journal.pone.0335405 (PMC12614528; doi:10.1371/journal.pone.0335405)
Supplement: S1 Table — P1 represents p-value of IPSA vs. MA; P2 represents p-value of HIPO vs. MA; P3 represents p-value of IPSA vs. HIPO. *: p-value≤0.05. All p-values were derived from paired t-tests and adjusted for multiple comparisons using the Benjamini-Hochberg (FDR) correction. (DOCX) [file pone.0335405.s001.docx]

S1 Table. Dosimetric comparison with the Benjamini-Hochberg false discovery rate correction of

MA, IPSA and HIPO plans in the ICBT cohort (mean ± SD)

|  | Parameter | MA | IPSA | HIPO | P1 | P2 | P3 |
| --- | --- | --- | --- | --- | --- | --- | --- |
| HRCTV | D_100_ (Gy) | 3.55±0.40 | 3.61±0.45 | 3.52±0.42 | 0.460 | 0.682 | 0.548 |
|  | V_150%_ (%) | 53.07±3.70 | 51.66±2.45 | 52.03±3.68 | 0.037**^*^** | 0.125 | 0.601 |
|  | V_200%_ (%) | 31.19±3.33 | 29.82±2.28 | 30.49±3.38 | 0.022**^*^** | 0.160 | 0.548 |
|  | HI | 0.41±0.04 | 0.43±0.03 | 0.42±0.04 | 0.037**^*^** | 0.125 | 0.601 |
|  | CI | 0.66±0.08 | 0.67±0.06 | 0.71±0.08 | 0.558 | 0.000**^*^** | 0.000**^*^** |
| Bladder | D_1cc_ (Gy) | 4.25±0.44 | 4.10±0.39 | 4.15±0.39 | 0.022**^*^** | 0.072 | 0.548 |
|  | D_2cc_ (Gy) | 3.94±0.40 | 3.83±0.38 | 3.84±0.37 | 0.030**^*^** | 0.055 | 0.646 |
| Rectum | D_1cc_ (Gy) | 3.37±0.72 | 3.25±0.69 | 3.29±0.72 | 0.022**^*^** | 0.125 | 0.548 |
|  | D_2cc_ (Gy) | 3.00±0.66 | 2.91±0.64 | 2.93±0.66 | 0.030**^*^** | 0.125 | 0.601 |
| Sigmoid | D_1cc_ (Gy) | 2.78±0.98 | 2.67±0.87 | 2.71±0.97 | 0.022**^*^** | 0.099 | 0.548 |
|  | D_2cc_ (Gy) | 2.48±0.86 | 2.39±0.79 | 2.41±0.85 | 0.022**^*^** | 0.072 | 0.601 |

P1 represents p-value of IPSA vs. MA; P2 represents p-value of HIPO vs. MA; P3 represents p-value of IPSA vs. HIPO.

*: p-value ≤ 0.05.

All p-values were derived from paired t-tests and adjusted for multiple comparisons using the Benjamini-Hochberg false discovery rate (FDR) correction.
