# Supplementary material for: Dosimetric divergence in ICBT vs. IC/ISBT configurations: Comparative analysis of three optimization algorithms for cervical cancer brachytherapy
Source: PLoS One. 2025 Nov 13;20(11):e0335405. doi: 10.1371/journal.pone.0335405 (PMC12614528; doi:10.1371/journal.pone.0335405)
Supplement: S2 Table — P1 represents p-value of IPSA vs. MA; P2 represents p-value of HIPO vs. MA; P3 represents p-value of IPSA vs. HIPO. *: p-value≤0.05. All p-values were derived from paired t-tests and adjusted for multiple comparisons using the Benjamini-Hochberg false discovery rate (FDR) correction. (DOCX) [file pone.0335405.s002.docx]

S2 Table. Dosimetric comparison with the Benjamini-Hochberg false discovery rate correction of

MA, IPSA and HIPO plans in the IC/ISBT cohort (mean ± SD)

|  | Parameter | MA | IPSA | HIPO | P1 | P2 | P3 |
| --- | --- | --- | --- | --- | --- | --- | --- |
| HRCTV | D_100_ (Gy) | 3.46±0.31 | 3.53±0.34 | 3.56±0.28 | 0.274 | 0.094 | 0.613 |
|  | V_150%_ (%) | 52.22±4.66 | 47.84±5.47 | 47.4±6.14 | 0.000**^*^** | 0.000**^*^** | 0.413 |
|  | V_200%_ (%) | 27.93±5.47 | 25.66±4.93 | 25.06±5.49 | 0.000**^*^** | 0.000**^*^** | 0.183 |
|  | HI | 0.42±0.05 | 0.47±0.06 | 0.47±0.07 | 0.000**^*^** | 0.000**^*^** | 0.413 |
|  | CI | 0.71±0.05 | 0.72±0.06 | 0.77±0.06 | 0.487 | 0.000**^*^** | 0.000**^*^** |
| Bladder | D_1cc_ (Gy) | 4.28±0.64 | 4.05±0.55 | 4.09±0.55 | 0.000**^*^** | 0.000**^*^** | 0.183 |
|  | D_2cc_ (Gy) | 3.96±0.57 | 3.80±0.52 | 3.81±0.52 | 0.000**^*^** | 0.000**^*^** | 0.613 |
| Rectum | D_1cc_ (Gy) | 3.99±0.72 | 3.80±0.65 | 3.75±0.69 | 0.647 | 0.000**^*^** | 0.000**^*^** |
|  | D_2cc_ (Gy) | 3.58±0.62 | 3.45±0.56 | 3.37±0.59 | 0.040**^*^** | 0.000**^*^** | 0.000**^*^** |
| Sigmoid | D_1cc_ (Gy) | 2.86±0.86 | 2.76±0.87 | 2.73±0.87 | 0.053 | 0.001**^*^** | 0.425 |
|  | D_2cc_ (Gy) | 2.55±0.79 | 2.47±0.79 | 2.44±0.78 | 0.065 | 0.001**^*^** | 0.413 |

P1 represents p-value of IPSA vs. MA; P2 represents p-value of HIPO vs. MA; P3 represents p-value of IPSA vs. HIPO.

*: p-value ≤ 0.05.

All p-values were derived from paired t-tests and adjusted for multiple comparisons using the Benjamini-Hochberg FDR correction.
